# Assessing Anxiety in Autistic and Non‐Autistic Youth: Validation of the German Parent Version of the Anxiety Scale for Children With Autism Spectrum Disorder

**DOI:** 10.1002/aur.70107

**Published:** 2025-08-21

**Authors:** Magdalena Gruner, Veit Roessner, Melanie Ring

**Affiliations:** ^1^ Department of Child and Adolescent Psychiatry, Medical Faculty Technische Universität Dresden, German Center for Child and Adolescent Health (DZKJ), Partner Site Leipzig/Dresden Germany

**Keywords:** anxiety, anxiety assessment, anxiety scale for children with autism, autism

## Abstract

Anxiety is a prevalent co‐occurring disorder in autistic youth, yet its accurate assessment remains challenging due to symptom overlap with autism. The Anxiety Scale for Children with Autism Spectrum Disorder—Parent Version (ASC‐ASD‐P) was designed to address this issue, but its utility in German clinical settings has not been established. This study validated the German translation of the ASC‐ASD‐P in a clinical sample of 317 participants presenting at a clinic for autism assessment, including 120 autistic youth. Internal consistency was excellent (Cronbach's *α* = 0.92), and convergent validity was demonstrated through significant correlations with established psychopathology measures such as the Child Behavior Checklist (CBCL) and Strengths and Difficulties Questionnaire (SDQ). Factor analyses preferred a 4‐factor structure in the autism group, but indicated difficulties replicating the Separation Anxiety Subscale. Autistic youth showed higher total anxiety and uncertainty scores compared to non‐autistic youth, underlining the scale's sensitivity to autism‐specific anxiety patterns. By including youth with intellectual disabilities, often underrepresented in research, this study provides a more comprehensive evaluation of the ASC‐ASD‐P's applicability across the autism spectrum. These findings support the ASC‐ASD‐P as a reliable tool for assessing anxiety in German‐speaking autistic youth while highlighting areas where refinement could strengthen its utility.

**Summary**

- Anxiety is a common concern for autistic youth, but it can be hard to identify.
- We tested a German version of a questionnaire called the Anxiety Scale for Children with Autism Spectrum Disorder—Parent Version (ASC‐ASD‐P) to see how well it can detect signs of anxiety.
- The questionnaire was reliable and good at identifying anxiety, especially anxiety related to uncertainty, but one part of the questionnaire measuring anxiety might need improvement.
- Parents of autistic youth rated their child's anxiety higher than parents of non‐autistic youth, showing that the questionnaire can capture autism‐specific anxiety.
- This tool can help parents and professionals better understand and support anxious autistic youth.

## 1 Introduction

Anxiety disorders are among the most prevalent co‐occurring disorders in autistic individuals, with 42% of autistic school‐aged children already presenting anxiety symptoms (Simonoff et al. 2008). Estimates of prevalence in youth vary widely, ranging from 22% to as high as 91%, but consistently demonstrate a higher risk of anxiety in autistic compared to non‐autistic youth (Vasa and Mazurek 2015; for meta‐analytic review see Van Steensel and Heeman 2017). Several theoretical frameworks help explain this elevated prevalence. Sensory processing difficulties, such as hypersensitivity to noise, touch, or light, are linked to intolerance of uncertainty and anxiety in autistic pre‐school youth (MacLennan et al. 2021). Research has shown that intolerance of uncertainty is also associated with increased anxiety in autistic youth and may mediate the link between autism spectrum disorder and anxiety symptoms (Boulter et al. 2014). Additionally, impairments in social skills—such as difficulties with perspective‐taking and interpreting social cues—can increase social anxiety and misunderstanding in interpersonal contexts (White et al. 2009). Together, these factors provide insight into the processes through which anxiety may emerge in autistic youth.

Critically, the correct detection of co‐occurring anxiety disorders in autistic youth is particularly challenging due to several factors. First, clinicians often struggle to determine whether symptoms should be considered as part of the autism or as a co‐occurring condition (Grondhuis and Aman 2012; White et al. 2009). For example, parents often notice an amplification of autistic characteristics when their child is anxious (Simpson et al. 2020). Or the anxiety symptoms may manifest themselves differently than in non‐autistic individuals (Belardinelli and Raza 2016). For example, autistic youth with co‐occurring obsessive‐compulsive disorder (OCD) exhibit fewer sexual obsessions, checking rituals, washing/cleaning rituals, and repetitive rituals compared to their non‐autistic counterparts with OCD (Lewin et al. 2011). Furthermore, Kerns et al. (2014) found that 46% of autistic youth in their study displayed anxiety symptoms that did not align with any Diagnostic and Statistical Manual of Mental Disorders (5th ed.; DSM–5; American Psychiatric Association 2013) diagnosis. These symptoms included excessive, yet specific worries, social fearfulness, rituals that are distressing but functionally unclear, and various unusual fears. According to Mayes et al. (2013), the three most frequently reported uncommon fears in autistic youth were fear of toilets, fear of elevators, and fear of vacuum cleaners. Another factor complicating the diagnosis of anxiety is that approximately one‐fifth of autistic youth also display intellectual disabilities (Khachadourian et al. 2023) which can contribute to the differential appearance of co‐occurring disorders (Belardinelli and Raza 2016). Parents and caregivers report lower anxiety levels in autistic youth with greater communication deficits. This may indicate that youth with severe communication deficits either experience lower levels of anxiety or, more plausibly, they face greater difficulties in expressing their emotions (Davis et al. 2011). This highlights the complex interplay of factors influencing anxiety and autism. Communication difficulties may also present a barrier for caregivers to identify symptoms of anxiety (Grondhuis and Aman 2012).

Anxiety has considerable negative consequences for autistic youth and their parents across all areas of daily life (Den Houting et al. 2022) and is related to a reduced quality of life (Adams et al. 2020; Van Steensel et al. 2012). It impairs youth's engagement and participation in home and community activities (Ambrose et al. 2022), disrupts peer relationships and academic performance (Adams and Emerson 2021). Moreover, Dadds et al. (1997) highlighted the importance of promptly treating anxiety symptoms. Their study found that 54% of youth showing symptoms of anxiety who were only monitored, but not treated, developed a full diagnosis of an anxiety disorder within 6 months. In addition, persistent anxiety and/or depression in youth has also been associated with reduced physical health including asthma, arthritis, heart problems, obesity, and sleep disturbances, as well as reduced daily functioning in adulthood, such as difficulties maintaining employment or education. These findings underscore the critical importance of early identification and treatment of anxiety disorders to mitigate their far‐reaching consequences (Morales‐Muñoz et al. 2023).

To mitigate the negative consequences of anxiety disorders, accurate identification is essential. This requires specialized tools that are both effective and feasible for use in everyday clinical practice. One such tool is the Anxiety Scale for Children with Autism Spectrum Disorder (ASC‐ASD, Rodgers et al. 2016), an autism‐specific anxiety questionnaire comprising 24 items. Developed in English, the ASC‐ASD is based on the Revised Children's Anxiety and Depression Scale (Chorpita et al. 2000), but includes additional items designed to capture more autism‐specific anxiety symptoms. The scale is available in both self‐report (child) and parent‐report (ASC‐ASD‐P) versions.

The ASC‐ASD has been validated in various languages and cultural contexts, including Farsi (Persian) in an Iranian sample (Samadi et al. 2020), English in a Singaporean sample (Soh et al. 2021) and Spanish in a Peruvian sample (Mikrukova and Rúa 2023). The Persian validation tested a sample of 254 parent reports and a subsample of 58 child self‐reports. It revealed good internal consistency and test–retest reliability, although parents tended to slightly underestimate their child's anxiety level, particularly on the Anxious Arousal Subscale, as indicated by the convergence of child and parent reports. Instead of the known four‐factor structure, only the three subscales Performance Anxiety, Uncertainty, and Anxious Arousal were identified (Samadi et al. 2020). The Spanish validation analyzed 91 child and parent reports. An exploratory factor analysis identified the three factors Uncertainty, Performance Anxiety, and Anxious Arousal. The fourth scale, Separation Anxiety, however, was again not replicated. Soh et al. (2021) validated the ASC‐ASD parent and child versions in a sample of 91 youths and their caregivers, reporting satisfactory to desirable internal consistency for all subscales, except Anxious Arousal of the parent version, as well as medium to large convergent validity. A limitation of these validation studies is that they included only youth with sufficient verbal abilities, limiting the generalizability of the findings. Autistic individuals with intellectual disabilities are often neglected in clinical studies (Russell et al. 2019) and very little is known especially about minimally verbal older youth (Tager‐Flusberg and Kasari 2013). To address this gap, the present study aims to examine a broad clinical sample with varying levels of intellectual functioning and co‐occurring psychiatric or neurodevelopmental conditions. This inclusive approach aims to capture the complexity of real‐world clinical presentations and allows for a more ecologically valid understanding of anxiety across the autism spectrum. By reflecting the heterogeneity seen in clinical practice, the study's findings may be more generalizable to the broader autistic population. Another important aspect to consider is that the cultural contexts of previous validations differ substantially in terms of underlying cultural values, particularly regarding individualism and collectivism. These dimensions can influence both the expression and self‐reporting of anxiety symptoms, potentially introducing cultural bias into item functioning or interpretation. In collectivist cultures, such as Iran and Peru, group harmony and the maintenance of interpersonal relationships are highly valued. Emotional restraint and control are often encouraged as a means of preserving social cohesion (Ryder et al. 2002). In contrast, in more individualistic cultures, such as those found in Western‐influenced parts of Singapore, personal experiences and internal states may be more openly expressed, as behavior tends to be guided by individual thoughts and feelings (Hofmann and Hinton 2014). These cultural variations underscore the importance of considering cross‐cultural differences when interpreting and comparing findings on anxiety symptoms across studies.

A further aim of the present study is to establish and validate a German version of the ASC‐ASD‐P for use in German‐speaking psychiatric contexts. Therefore, we assess the reliability of the German version and examine its validity by investigating its underlying factor structure and evaluating its convergent validity by examining the relationship between the ASC‐ASD‐P and other measures of psychopathology commonly used in clinical contexts. It is essential to investigate the factor structure of the German version of the ASC‐ASD‐P in order to determine whether the underlying construct can be replicated in the German context. While earlier validations have demonstrated promising psychometric properties, cultural and linguistic differences may influence how items are understood and how anxiety symptoms manifest. Examining the factor structure in a German‐speaking sample ensures that the translated scale maintains its construct validity and functions as intended. This analysis will also help establish whether the theoretical dimensions of the original scale are applicable in the German cultural and clinical context, thereby supporting its reliable use in both research and practice. Standardized instruments such as the DYSIPS‐III (Döpfner and Görtz‐Dorten 2017), the Child Behavior Checklist (CBCL/4‐18, Döpfner et al. 1994), and the Strengths and Difficulties Questionnaire (SDQ, Goodman 1997) are commonly used for the assessment of anxiety in clinical settings in Germany and are valid for most clinical and research purposes (Klasen et al. 2000). However, these tools were not specifically developed or validated for use in autistic populations (Halvorsen et al. 2024) and may therefore lack sensitivity to the distinct and sometimes atypical presentation of anxiety in autism (Kerns et al. 2021). Given these limitations, our study aims to contribute to the availability of autism‐specific assessment tools by validating a German version of the ASC‐ASD‐P, thereby supporting more accurate and clinically relevant identification of anxiety in autistic youth.

## 2 Methods

### 2.1 Participants

A total of 327 youth and their parents participated in this study. Ten participants were excluded from the analyses because of too many missing values in the ASC‐ASD‐P questionnaire, resulting in a final sample of 317 participants (120 autistic and 197 non‐autistic youth, attrition rate = 3.1%). The sample size was calculated to ensure adequate power for conducting confirmatory factor analyses. Sample size recommendations were based on the guidelines provided by Comrey and Lee (2013) and Bühner (2011), who suggest a minimum of *N* = 200 for an adequate sample size and *N* = 300 for a good sample size. Additionally, Nunnally and Bernstein (2010) recommend a participant‐to‐item ratio of 10:1, which corresponds to a minimum sample size of *N* = 240 for the 24 items of the ASC‐ASD‐P. Recruitment took place between September 2021 and May 2024. Participating families either completed the questionnaires as part of the routine diagnostic assessments of youth at the Autism outpatient clinic of the University hospital Carl Gustav Carus Dresden (*N* = 221) or they participated in a larger study (*N* = 96) investigating a model of anxiety in autistic youth. Prior to study participation, all participants underwent screening. Questionnaires were completed by the youth's parents or another close relative or caregiver (e.g., grandparents).

The autism group consisted of youth with a clinical diagnosis of Childhood Autism (F84.0), Atypical Autism (F84.1) or Asperger Syndrome (F84.5) according to the International Classification of Diseases 10 (ICD‐10, World Health Organization 1993). Sixteen participants had another Pervasive Developmental Disorder (F84). Diagnoses were established using the diagnostic gold standard, including the German Autism Diagnostic Interview‐Revised (ADI‐R; Bolte et al. 2006) and the German Autism Diagnostic Observation Schedule (ADOS‐2; Poustka et al. 2015; Rühl et al. 2004). Co‐occurring diagnoses were not an exclusion criterion for either group. In the autism group, 45 participants (37.5%) had no co‐occurring psychiatric disorder, while 57 (47.5%) had one, and 18 (15%) had two or more. Twelve (10%) participants had an IQ below 70, while 8 (6.7%) exhibited a cognitive age delay of at least 24 months on the Bayley‐III. The non‐autism group included youth who either presented at the Autism outpatient clinic for diagnostic evaluation but did not receive an autism diagnosis, or who participated in the larger study in the non‐autism control group. In 70 youth from the non‐autism group, autism was suspected but neither confirmed nor ruled out. In the non‐autism group, 101 (51.3%) participants had no psychiatric disorder, while 49 (24.9%) had one, and 47 (23.9) had two or more. Ten participants (5.7%) had an IQ below 70, while 6 (3%) exhibited a cognitive age delay of at least 24 months on the Bayley‐III.

Autistic and non‐autistic youth did not differ in age, *t*(256.9) = 0.19, *p* = 0.852, *d* = 0.02. However, the groups differed with respect to biological sex, *χ* ^*2*^(1, *N* = 317) = 4.74, *p =* 0.029, φ = 0.01. Only approximately one‐quarter of the autism group were girls, compared to one‐third of the non‐autism group. This corresponds with existing research indicating that autism is diagnosed three to four times more often in males than in females (Loomes et al. 2017; Posserud et al. 2021), and aligns with the demographic composition of our sample. Full‐scale IQ was lower in the autism group than in the non‐autism group, *t*(114.6) = −2.57, *p* = 0.011, *d* = 0.42, but there was no difference in cognitive developmental age, *t*(39.40) = 0.30, *p* = 0.764, *d* = 0.09. For detailed information about the sample see Table 1.

**TABLE 1:** Descriptive data of the sample.

	Autism group (*N* = 120)		Non‐autism group (*N* = 197)	
Gender	Male = 97, female = 23		Male = 136, female = 61	
	*M*	SD	*M*	SD
Age	10.0	4.4	9.9	4.5
Full‐Scale IQ	88.0	21.8	96.3	18.3
Bayley‐III				
Cognitive developmental age	51.7	19.6	48.8	25.3
Age	68.9	17.4	62.5	20.8
ADOS‐2			—	
Social interaction	10.8	4.2		
Stereotyped behaviors, restricted interests	3.1	2.7		
Total score	13.7	5.1		
Total score range	3–26			
ADI‐R			—	
Social interaction	17.2	5.5		
Communication	12.5	4.4		
Repetitive behavior	4.7	2.6		
Autism diagnoses	Number of cases		Number of cases	
F84.0	57		—	
F84.1	14			
F84.5	33			
Co‐occurring disorders				
F10–F19	0		1	
F12	0		1	
F30–F39	1		6	
F32	0		6	
F33	1		0	
F40–F48	2		22	
F40	0		7	
F41	2		3	
F42	0		3	
F43	0		8	
F45	0		1	
F50–F59	1		1	
F50	1		1	
F60–F69	1		2	
F60	0		1	
F63	1		1	
F70–F79	12		12	
F70	6		7	
F71	1		1	
F74	5		4	
F80–F89	20		13	
F80	5		3	
F81	0		3	
F82	11		1	
F83	1		3	
F84	3		2	
F85	0		1	
F90–F98	38		102	
F90	21		27	
F91	0		4	
F93	1		27	
F94	1		4	
F95	0		5	
F98	15		36	

*Note*: ADOS‐2 total score represents the sum of the subscales Social Interaction and Stereotyped behaviors for ADOS‐2 Modules 1–3 and the sum of the subscales Communication and Social interaction for Module 4. ADOS‐2 was not available for 13 youth. Full‐scale IQ was measured with a Wechslertest (WISC, WAIS, WPPSI, HAWIKW, WNV) or the K‐ABC‐II, SON‐R, or the ZVT (Trail‐making test). Bayley‐III cognitive and developmental age are reported in months. IQ data were available for 85 autistic youth and 124 non‐autistic youth.

### 2.2 Procedure

The study was approved by the ethics committee of TU Dresden (ethical approval code: EK 356092018) and conducted in accordance with the principles of the Declaration of Helsinki. Written informed consent was obtained from all participating youths and their parents or legal guardians. Participation involved the completion of several questionnaires, which the respective informants filled out at home. The questionnaires were administered either in paper format or online via LimeSurvey (Limesurvey GmbH, n.d.). The relevant questionnaires were: The German version of the ASC‐ASD‐P (Rodgers et al. 2016), the German AQ‐Child/Adol version (Baron‐Cohen et al. 2006), the German Child and Behavior Checklist (CBCL/4‐18, Achenbach 1991) and the German version of the Strength and Difficulties Questionnaire (SDQ, Goodman 1997). Data from the additional questionnaires (AQ‐Child/Adol, CBCL and SDQ) was available only for a subsample of 284 youths (110 autistic and 174 non‐autistic youths).

### 2.3 Measures

#### 2.3.1 Anxiety Scale for Children With Autism Spectrum Disorder—Parent Version (ASC‐ASD‐P)

The ASC‐ASD‐P is a 24‐item questionnaire to assess autism‐specific anxiety symptoms. The items can be grouped into four subscales: Performance Anxiety (5 items), Anxious Arousal (6 items), Separation Anxiety (5 items) and Uncertainty (8 items). Each item is rated on a 4‐point Likert scale (ranging from never to always), with higher scores indicating higher levels of anxiety. A total Anxiety score can be calculated by summing all items, in addition to four subscale scores. The original English version showed good to excellent internal consistency (parent version: Cronbach's alpha = 0.94 for the total score and 0.87–0.91 for the subscales) and test–retest reliabilities (parent version: *r* = 0.84; Rodgers et al. 2016). A total score of 20 is recommended as the cut‐off point for clinically significant anxiety symptomatology.

#### 2.3.2 Autism Spectrum Quotient—Adolescent Version (AQ‐Child/Adol)

The AQ is a validated 50‐item questionnaire developed to quantify autism‐like traits in adults (Baron‐Cohen et al. 2001). The German AQ‐Child/Adol is a combination of the original parent‐report adaptation for adolescents aged 12–15 years by Baron‐Cohen et al. (2006) and the parent‐report adaptation for children aged 4–11 years (Auyeung et al. 2008). The questionnaire assesses five different domains, each comprising 10 items: Social Skills, Attention Switching, Attention to Detail, Communication, and Imagination. Responses are provided on a 4‐point Likert scale, ranging from 0 = “definitely agree”, 1 = “slightly agree”, 2 = “slightly disagree” to 3 = “definitely disagree”, following the scoring approach commonly employed in recent studies (Hoekstra et al. 2008). The total AQ‐Child/Adol score is calculated by summing all item scores, yielding a range from 0 to 150, where higher scores indicate greater autism‐like traits. Although not a diagnostic tool, the AQ‐Child/Adol can serve as a screening instrument for autism, with a total score of 76 suggested as a cut‐off score (Auyeung et al. 2008). Cronbach's Alpha for the current study indicated excellent internal consistency (*α* = 0.92).

#### 2.3.3 Child Behavior Checklist (CBCL/4‐18)

The CBCL/4‐18 is a validated instrument designed to evaluate parental perceptions of their child's behavioral‐emotional problems and competencies (Achenbach 1991). While the questionnaire was updated (CBCL/6‐18) in 2001 (Achenbach and Rescorla 2001), the German translation of the CBCL/4‐18 (Döpfner et al. 1994) was utilized in this study due to the age range of the sample. The first section focuses on adaptive behaviors, whereas the second assesses problem behaviors over the past 6 months. For the purpose of this study, only items assessing problem behaviors were used. Items are scored on a scale from 0 (not true) to 2 (very true). The items assessing problem behaviors can be used to calculate eight narrow subscales (Rule Breaking, Aggressive Behavior, Withdrawn‐Depressed, Somatic Complaints, Anxious‐Depressed, Social Problems, Thought Problems and Attention Problems), two broader scores (Internalizing and Externalizing) and a total Behavior Problems Score. For this study, only the Anxious‐Depressed subscale and the Internalizing score were considered for evaluating the convergent validity of the ASC‐ASD‐P. They showed good to excellent internal consistency in the German version, and the factor structure of the English version was largely replicated (Döpfner et al. 1994).

#### 2.3.4 Strengths and Difficulties Questionnaire (SDQ)

The SDQ is a behavioral screening tool comprising 25 items that assess both positive and problematic behaviors. The items are organized into five subscales with five items each: Conduct Problems, Hyperactivity‐Inattention, Emotional Symptoms, Peer Problems, and Prosocial Behavior (Klasen et al. 2000). Respondents are asked to evaluate the child's behavior over the past six months, rating items on a scale from 0 (not true) to 2 (certainly true). A total difficulties score can be calculated by summing the scores of the four subscales: Hyperactivity, Emotional Symptoms, Conduct Problems, and Peer Problems. Only the subscale Emotional Symptoms is relevant for this study. Originally developed in English by Goodman (1997), the German translation of the SDQ was employed in its parent and teacher version for children aged 4 to 17 years. The German translation replicated the factor structure of the original version and demonstrated satisfactory to good reliability, with Cronbach's Alpha ranging from 0.58 to 0.76 (Woerner et al. 2002).

### 2.4 Statistical Analyses

Missing data were handled as follows: For the ASD‐ASD‐P, a maximum of one item per scale and four items total could be missing and were replaced with the mean score of the respective subscale in accordance with Rodgers et al. (2016). For the AQ‐child/adol, a corrected total score was computed if less than five items were missing by following the procedure suggested by Hoekstra et al. (2008) and Auyeung et al. (2008): total AQ‐child/adol score + (mean item score × number of missing items). The CBCL was allowed to have up to two missing items per scale and up to eight missing items for the computation of the broad index scales, Internalizing, Externalizing, and Total Problems. Finally, for the SDQ, when at least four items per scale were answered, the mean of the scale was calculated, and the total score was estimated when at least 16 items total were completed. All questionnaires with more missing items than the outlined numbers were considered incomplete and discarded from analyses (ASC‐ASD‐P: *N* = 10; CBCL: *N* = 18, SDQ: *N* = 3). In cases where SDQ and CBCL were answered separately by mothers and fathers, the average score of both parent reports was computed.

Reliability was assessed using Cronbach's Alpha. To evaluate split‐half reliability, the items were divided into odd and even groups, and their correlation was assessed, with Spearman–Brown coefficients reported. Descriptive analyses included independent sample t‐tests of the total and subscale scores for each questionnaire. Corrections for multiple comparisons were applied using the Holm method. Convergent validity of the ASC‐ASD‐P was explored via Pearson correlational analyses between the ASC‐ASD‐P and the AQ‐Child/Adol, CBCL, and SDQ total scores, as well as their respective subscale scores measuring internalizing problems. Group differences in the correlations between ASC‐ASD‐P and AQ‐Child/Adol, CBCL, and SDQ were explored using Fisher r‐to‐Z transformation. Confirmatory factor analysis (CFA) was conducted with weighted least squares means and variance adjusted estimation (WLSMV) to examine the internal structure of the ASC‐ASD‐P. The CFA model included the four‐factor structure of the ASC‐ASD‐P, consisting of Performance Anxiety, Separation Anxiety, Anxious Arousal, and Uncertainty. No modifications were applied to the initial model. However, exploratory factor analyses were subsequently conducted to further investigate the factor structure. CFA fit indices were interpreted based on the criteria proposed by Hu and Bentler (1999) who suggested the following cut‐off scores as indicators of good model fit: Comparative fit index (CFI) and Tucker–Lewis index (TLI) > 0.95, Standardized Root Mean Square Residual (SRMR) < 0.09, and Root Mean Square of Error Approximation (RMSEA) < 0.06. All statistical analyses were performed using R (R Core Team 2023, version 4.3.2). A significance level of *p* < 0.05 was applied for all analyses, and Cohen's *d* was reported as effect size for between‐group comparisons.

## 3 Results

### 3.1 Group Comparisons

Total ASC‐ASD‐P scores were higher in the autism group compared to the non‐autism group. At a subscale level, significant group differences were found only for the Uncertainty subscale of the ASC‐ASD‐P, with higher scores observed in the autism group compared to the non‐autism group (see Table 2).

**TABLE 2:** Questionnaire scores in the autism and non‐autism groups.

	Autism group		Non‐autism group		Comparison			
	*M*	SD	*M*	SD	*t*	df	*p*	*d*
ASCASD‐P total score	21.4	12.9	18.3	13.4	2.02	258.1	0.045	0.23
ASC‐ASD‐P subscale scores								
Separation anxiety	4.5	3.7	4.0	3.6	1.20	248	0.696	0.14
Performance anxiety	3.9	3.8	4.0	3.8	‐ 0.27	252.3	0.922	0.03
Anxious arousal	2.2	2.3	2.0	2.6	0.74	270.7	0.922	0.08
Uncertainty	10.8	6.3	8.3	6.7	3.31	265.1	0.004	0.38
AQ total score	95.7	16.7	80.4	28.7	5.41	255.9	< 0.001	0.62
AQ subscale scores								
Social skill	20.7	5.2	17.4	7.9	4.04	259.9	< 0.001	0.47
Attention switching	21.1	4.9	17.5	7.2	4.82	259.9	< 0.001	0.56
Attention to detail	14.3	6.1	13.3	5.8	1.31	213.2	0.192	0.17
Communication	20.8	4.6	17.1	7.5	4.92	258	< 0.001	0.57
Imagination	18.6	4.9	15.0	6.2	5.32	253.1	< 0.001	0.64
CBCL total score	55.4	22.4	47.4	29.9	2.56	273.4	0.011	0.29
CBCL subscale anxious‐depressed	5.8	4.7	5.2	4.8	0.95	234.7	1.00	0.12
CBCL broad subscale Internalizing	14.1	8.0	12.1	9.2	1.89	254.4	0.353	0.22
SDQ total score	17.3	5.1	14.6	7.7	3.50	281.7	< 0.001	0.39
SDQ subscale emotional symptoms	3.07	2.3	2.8	2.4	0.90	238.1	0.742	0.11

*Note: p*‐values for subscale comparisons are Bonferroni–Holm corrected.

In the autism group, 50% of youth and 41% of the non‐autism group met the primary anxiety cut‐off of the ASC‐ASD‐P (total score ≥ 20). The secondary cut‐off (total score ≥ 24) was met by 41% of the autism group and 30% of the non‐autism group. Across both groups and the entire sample, the most endorsed item was Item 16 of the Subscale Uncertainty (for details on item meanings see Table S8), with 78.9% of parents in the entire sample (85.0% autism and 75.1% non‐autism‐group) reporting that the statement was at least sometimes true for their child. The most endorsed subscale was Uncertainty, followed by Separation Anxiety, Performance Anxiety, and Anxious Arousal in both the entire sample and the autism group. In the non‐autism group, the most endorsed subscale was Uncertainty, followed by Performance Anxiety, Separation Anxiety, and Anxious Arousal. Subscale endorsement differences were statistically significant, except for the difference between Separation Anxiety and Performance Anxiety (for more detail see Table S1).

Total AQ‐Child/Adol parent‐report scores, as well as subscale scores, were higher in the autism group than in the non‐autism group, with the exception of the Subscale Attention to Detail (see Table 2), where no between‐group differences were observed. Significant group differences were found on all other scales.

Total CBCL parent‐report scores were higher in the autism group compared to the non‐autism group (see Table 2 for relevant subscales). Subscale scores were higher for the subscales Withdrawn‐Depressed, Social Problems, and Attention Problems in the autism group compared to the non‐autism group. No between‐group differences were observed for the remaining subscales (see Table S2).

Total SDQ parent‐report scores were also higher in the autism group than in the non‐autism group (see Table 2). Scores for the subscales Hyperactivity‐Inattention and Peer Problems were higher in the autism group, while the non‐autism group scored higher on the subscale Prosocial Behavior. No between‐group differences were observed for the remaining subscales (see Table S3).

### 3.2 Correlational Analyses

**FIGURE 1:**
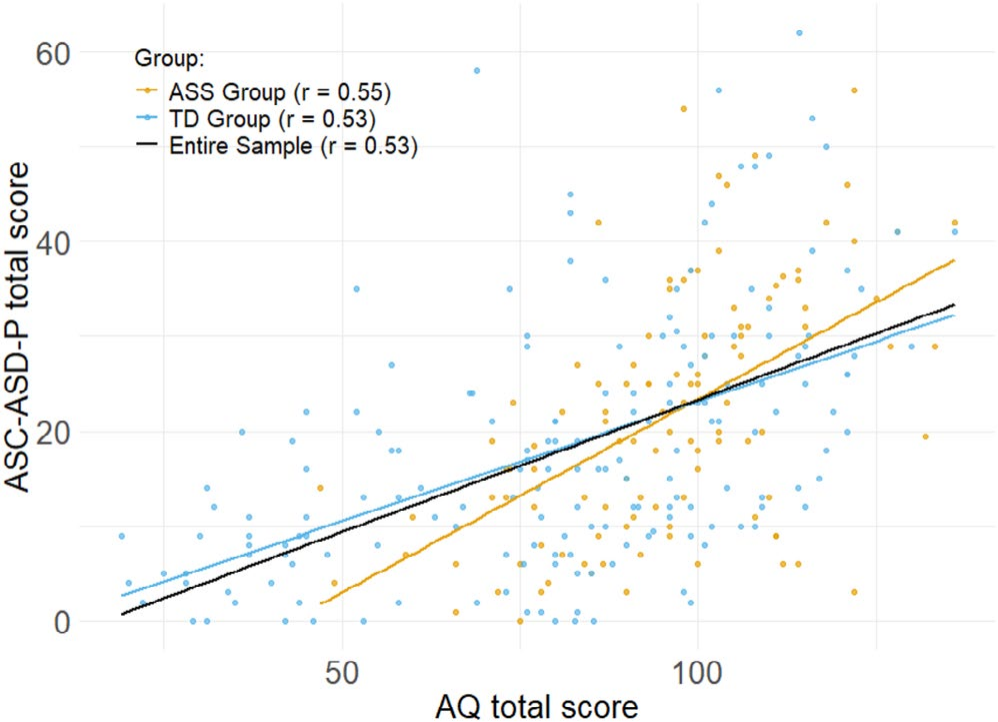
Correlation between AQ and ASC‐ASD‐P total scores by group.

A strong positive correlation was observed between ASC‐ASD‐P and AQ‐Child/Adol total scores in the entire sample and within both groups (all *p* < 0.001; see Figure 1). No differences in correlation strength were identified between the groups; *z* = 0.28, *p* = 1.00, *q* = 0.029 (Holm‐corrected *p*‐value). At the subscale level, the strongest correlation was found for the subscale Attention Switching (*r* = 0.63; 0.61; 0.63 in the entire sample, autism‐ and non‐autism‐groups, respectively, for more information see Table S4).

**FIGURE 2:**
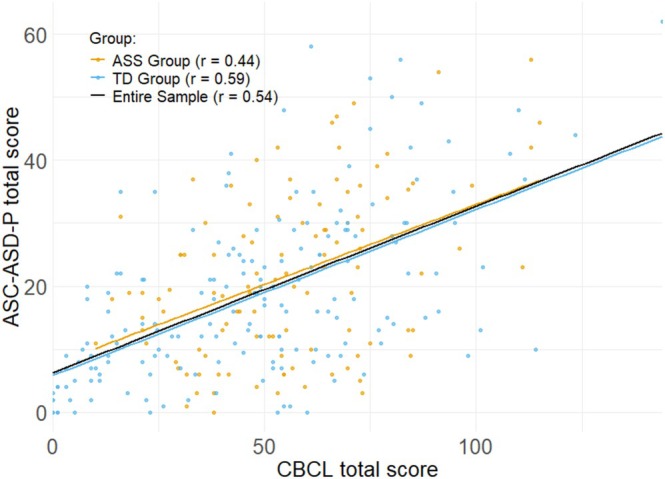
Correlation between CBCL and ASC‐ASD‐P total scores by group.

A strong positive correlation was also observed between ASC‐ASD‐P and CBCL total scores in the entire sample and in the non‐autism group, while the correlation was moderate in the autism group (all *p* < 0.001; see Figure 2). A small, non‐significant difference in correlation strength was observed between the autism and non‐autism groups, *z* = −1.97, *p* = 0.075, *q* = −0.20. At the subscale level, the strongest correlations were within the subscale Anxious‐Depressed (*r* = 0.71; 0.63; 0.76 in the entire sample, autism‐group and non‐autism‐group, respectively) and the Internalizing score (*r* = 0.66; 0.58; 0.69 in the entire sample, autism and non‐autism group, respectively).

**FIGURE 3:**
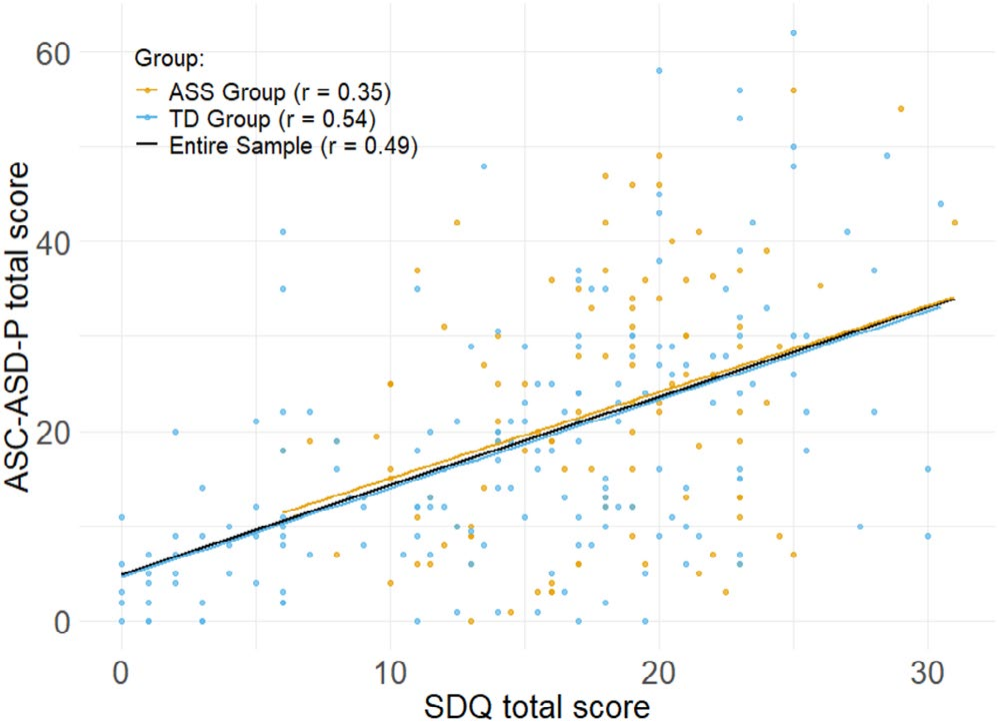
Correlation between SDQ and ASC‐ASD‐P total scores by group.

For the SDQ, a moderate to strong positive correlation with ASC‐ASD‐P total scores was observed in the entire sample and the non‐autism group, with a moderate correlation in the autism group (all *p* < 0.001; see Figure 3). This correlation was slightly stronger in the non‐autism group than in the autism group, *z* = −2.27, *p* = 0.036, *q* = −0.23. At the subscale level, the strongest correlation was found for the subscale Emotional problems (*r* = 0.71; 0.65; 0.75 in the entire sample, autism and non‐autism group, respectively).

### 3.3 Reliability of the ASC‐ASD‐P

At the item level, the ASC‐ASD‐P exhibited excellent internal consistency, with a Cronbach's Alpha of 0.92 for the entire sample and for both groups. Means of the corrected item‐total correlations (CITC) were 0.55, 0.54, and 0.56, respectively. However, CITCs < 0.3 were observed for Item 22 on the subscale Anxious Arousal in the entire sample and the autism group, whereas no items showed CITCs < 0.3 in the non‐autism group. At a subscale level, Cronbach's Alpha ranged from 0.77 to 0.91 for the entire sample, 0.74 to 0.89 in the autism group, and 0.76 to 0.92 in the non‐autism group. The mean of the corrected subscale‐total correlations (CSTC) was 0.61 for the entire sample and the autism group, and 0.60 in the non‐autism group. No CSTCs fell < 0.3 in either group. Split‐Half‐Reliability showed correlations of 0.96 for the entire sample, 0.95 in the autism group, and 0.96 in the non‐autism group.

### 3.4 Factor Structure of the German ASC‐ASD‐P

CFAs were performed to examine a 4‐factor model corresponding to the subscale structure of the original ASC‐ASD‐P for the entire sample and both groups. The items had a mean standardized regression weight of 0.80 for the entire sample (0.60–0.92), 0.78 in the autism group (0.39–0.91) and 0.81 in the non‐autism group (0.67–0.97, for detailed description of the factor loadings see Table S8).

Table 3 contains the fit indices for the factor analyses for the entire sample and both groups. Overall, results were mixed. While the chi‐square tests yielded significant *p*‐values, the relative chi‐square values remained below 5. CFI and TLI indicated good model fit, while RMSEA and SRMR indicated poor model fit for the entire sample and both groups. In summary, the data indicate that the model fit was not optimal in some regards.

**TABLE 3:** Fit indices of the confirmatory factor analyses by group.

Group	*χ* ^*2*^	df	*χ* ^*2*^/df	*p*	CFI^a^	TLI^b^	RMSEA^c^	SRMR^d^
Original 4‐factor structure								
Entire sample	815.7	246	3.32	< 0.001	0.98	0.98	0.086 [0.079, 0.092]	0.101
Autism group	411.3	246	1.67	< 0.001	0.98	0.98	0.075 [0.062, 0.088]	0.112
Non‐autism group	664.9	246	2.70	< 0.001	0.98	0.97	0.093 [0.085, 0.102]	0.116

^a^ Comparative Fit Index.

^b^ Tucker–Lewis Index.

^c^ Root Mean Square of error approximation, 90% confidence intervals in brackets.

^d^ Standardized root mean square residual.

To further investigate the factor‐structure of the German ASC‐ASD‐P, exploratory factor analyses were performed. The model preferred five factors in the entire sample and four factors when considered separately for each group. The subscale Separation Anxiety could not be replicated (for detailed description see Supporting Information, page no. 7–8).

## 4 Discussion

This study aimed to validate the German translation of the parent‐reported Anxiety Scale for Children with Autism Spectrum Disorder (ASC‐ASD‐P) in a large clinical sample comprising both autistic and non‐autistic youth. The results revealed that 50% of youth in the autism group exhibited clinically elevated anxiety levels, as indicated by the ASC‐ASD‐P's primary cut‐off. This rate is lower than the 75% reported by Den Houting et al. (2019). However, estimates of anxiety prevalence in autistic youth vary substantially (Vasa and Mazurek 2015), with recent meta‐analyses suggesting that only approximately one‐third of autistic youth experience clinically elevated levels of anxiety (Thiele‐Swift and Dorstyn 2024).

Regarding the prevalence of anxiety in autistic youth, comparison of the ASC‐ASD‐P scores between the present autism sample and the Singaporean sample of Soh et al. (2021) showed similar total scores (*t*(199.8) = 1.36, *p* = 0.697) and scores on the Anxious Arousal subscale (*t*(189.5) = 0.00, *p* = 1.00). However, scores on the Performance Anxiety Subscale were higher in Soh et al.'s sample (*t*(188.6) = −4.04, *p* = < 0.001), whereas higher scores were observed in our sample for Separation Anxiety (*t*(208) = 2.60, *p* = 0.040) and Uncertainty (*t*(1207.288.6) = 4.09, *p* = < 0.001). The ASC‐ASD‐P total score in the original sample of the study by Rodgers et al. (2016) was significantly higher than in our sample (*t*(274.6) = −2.96, *p* = 0.003). These variations may reflect differences in sample characteristics, such as age, cultural context, intellectual abilities, and clinical background. For instance, the youth in our sample were younger and had a broader age range (*M* = 10 years, range 3–19 years) compared to the Singaporean sample by Soh et al. (*M* = 14 years, range 9–18 years). Younger children tend to report higher levels of separation anxiety than older children (Birmaher et al. 1997), which could contribute to the higher Separation Anxiety scores in this study. According to the Organization for Economic Co‐operation and Development's (OECD) Program for International Student Assessment (PISA) report, 76% of students in Singapore reported that they compete with their peers, compared to only 33% in Germany. Similarly, 72% of Singaporean students expressed fear of failure, in contrast to 48% in Germany (OECD 2019a, 2019b, 2019c). These cultural factors may explain variations in Performance Anxiety. In general, cultural context shapes both the manifestation of anxiety symptoms and their clinical interpretation (Marques et al. 2014). Keating et al. (2024) demonstrate that autistic individuals across diverse countries—including Japan, New Zealand, Belgium, and South Africa—report varying levels of autism acceptance, camouflaging behaviors, and co‐occurring mental health challenges such as anxiety and depression. These findings underscore the importance of considering cultural influences in understanding anxiety among autistic youth, and highlight the need for future research to explore these dynamics in greater depth.

Consistent with the findings of Soh et al. (2021), the Uncertainty subscale was the most endorsed in both groups. However, autistic youth scored higher than non‐autistic youth only on this subscale, highlighting this subscale's particular sensitivity to anxiety patterns specific for autism. This distress due to a perceived lack of essential information, known as Intolerance of Uncertainty (IU; Carleton 2016) tends to be more elevated in autistic youth compared to typically developing youth (Boulter et al. 2014; Chamberlain et al. 2013; Vasa et al. 2018). Meta‐analytic reviews have shown a strong correlation between IU and anxiety in autistic individuals in general (Jenkinson et al. 2020) and specifically in autistic youth (Osmanağaoğlu et al. 2018). Our findings thus underscore the importance of IU as a critical mechanism linking uncertainty to anxiety in autism. This theoretical insight suggests that IU may serve as a focal point for anxiety interventions in autistic youth. For example, the “Coping with Uncertainty in Everyday Situations” (CUES; Rodgers et al. 2017, 2019) is an 8‐week program helping parents of autistic youth teach tolerance for everyday uncertainty. A feasibility trial confirmed its practicality for parents and therapists (Rodgers et al. 2023).

Correlational analyses revealed moderate to strong correlations between ASC‐ASD‐P total scores and subscales measuring internalizing symptoms on both the CBCL and SDQ. Specifically, the ASC‐ASD‐P strongly correlated with the emotional subscale of the SDQ across all groups (entire sample: *r* = 0.71; autism group: *r* = 0.65; non‐autism group: *r* = 0.75), aligning with the correlation of *r* = 0.76 reported in the original sample by Rodgers et al. (2016). Spence et al. (2003) also found moderate to strong correlations between the ASC‐ASD‐P and the Spence Children's Anxiety Scale (SCAS; Spence et al. 2003). These findings suggest that the German version of the ASC‐ASD‐P demonstrates strong convergent validity and aligns well with established measures of child psychopathology, supporting its suitability as an anxiety assessment tool (see also Den Houting et al. 2018; Keen et al. 2019).

Further, the ASC‐ASD‐P showed a stronger correlation with the AQ‐Child/Adol total score in our autism sample (*r* = 0.55) compared with Soh et al. (2021) (*r* = 0.40). While correlations with the AQ‐Child/Adol subscales exhibited some variation across studies, they were descriptively higher in our sample overall (see Table S5). Notably, the majority of autistic youth in this study had received their autism diagnosis very recently, following the completion of the questionnaires. Thus, both anxiety symptoms and autism‐like traits, as assessed by the AQ, may be more pronounced, as these youth have not yet had access to adequate treatment or effective coping strategies to manage their symptoms. Notably, the strongest correlation observed in both studies was found for the AQ subscale Attention Switching. Due to this strong association, Attention Switching may serve as a potential indicator of increased risk for anxiety. However, this association needs to be explored further in future research to allow for more precise clinical implications. Anxious youth with lower attention shifting ability showed higher anxiety symptoms compared to those with a higher ability to shift their attention (Ramos et al. 2022). These findings highlight the importance of understanding how cognitive difficulties like reduced attention switching contribute to emotional outcomes in autistic youth. Against this background, the observed reduced efficacy of Cognitive‐Behavioral Therapy (CBT)—particularly in addressing anxiety‐related life interference—in autistic youth with a higher degree of autism traits (Cervin et al. 2023) can be understood more clearly. Autism‐related characteristics, including cognitive rigidity, may potentially act as barriers to the success of standard CBT approaches, which may not sufficiently address the core cognitive inflexibility in autistic youth. Developing tailored interventions to address the unique challenges associated with high autism traits, such as adaptation to daily changes, may enhance treatment effectiveness. Another important clinical implication of our study is that interventions should consider autism‐specific anxiety‐eliciting situations identified by the questionnaire. For instance, the need for routines and predictability, as reflected in items 16, 20, and 23, should be addressed by providing structured environments or flexibility within routines to reduce anxiety. Additionally, interventions should account for sensory sensitivities, which can significantly increase anxiety, as highlighted by items 5 and 21. Tailored strategies, such as sensory integration techniques (Pfeiffer et al. 2011), could help individuals better manage these anxiety‐provoking triggers and enhance overall treatment outcomes.

Reliability, as indicated by Cronbach's Alpha, showed excellent internal consistency for the entire questionnaire, with subscale reliabilities ranging from acceptable to excellent. In terms of construct validity, the fit indices of the CFA yielded mixed results, making definite conclusions about the factor structure of the German ASC‐ASD‐P challenging. Exploratory factor analyses (EFA) provided somewhat inconclusive results regarding the number of factors, with a 4‐factor solution emerging as the preferred model for the autism group. Notably, Items 19 and 24 appeared problematic, as they did not load onto the original Separation Anxiety Subscale. Revising these items or excluding them from the German translation might improve the questionnaire's factor structure. This difficulty in replicating the Separation Anxiety subscale has also been observed in other translated versions of the ASC‐ASD‐P (Mikrukova and Rúa 2023; Samadi et al. 2020). While separation anxiety might be perceived as less relevant in autistic youth, it is crucial to acknowledge that its manifestations in this population may diverge from those observed in neurotypical individuals. As previously mentioned, a different presentation of anxiety in autistic youth through interaction with autistic traits is common (Kerns et al. 2014). Differences in sensory processing or communication styles may obscure traditional indicators of social interaction‐related forms of anxiety, such as separation anxiety. Conventional assessment tools, tailored for neurotypical behaviors, may not adequately capture these atypical presentations, potentially resulting in an underestimation of separation anxiety among autistic individuals. Consequently, our findings may reflect the limitations inherent in current assessment methodologies, including the ASC‐ASD‐P, rather than indicating a genuine absence of separation anxiety in autistic youth.

Several limitations of this study should be acknowledged. Firstly, the non‐autism sample included youth with suspected autism diagnoses, which complicates the interpretation of differences between the autism and non‐autism groups, as this sample does not represent a typical comparison group. Additionally, the presence of co‐occurring disorders like attention‐deficit hyperactivity disorder or intellectual disabilities in both groups may have an impact on the results. Notably, some youth in the non‐autism group were diagnosed with an anxiety disorder, which may account for the limited differences in anxiety levels observed between the two groups on the ASC‐ASD‐P, where effect sizes were relatively small. Studies comparing autistic and typically developing youth might find larger differences in anxiety scores than the present study. However, given that over 70% of autistic youth have at least one co‐occurring psychiatric disorder (Simonoff et al. 2008), including such individuals offers a more naturalistic representation of the clinical population. It should also be acknowledged that 16.7% of autistic participants in our sample had an IQ level below 70 or a cognitive delay of at least 24 months, and the mean full‐scale IQ was significantly lower in the autism group compared with the non‐autism group. Prior research suggests that autistic youth with intellectual disabilities may exhibit anxiety in ways that differ from DSM‐defined presentations (Kerns et al. 2021), and cognitive ability can influence both the expression and perception of anxiety symptoms. As our analyses focused on group‐level comparisons, potential variation related to cognitive functioning within the autism group may not be fully captured and could be examined in more detail in further research. Future work may specifically investigate whether factor structures vary across different cognitive profiles within autistic populations. Another limitation is the potential for recall bias in parent‐reported data. It can be challenging for parents to accurately report on their children's internal thoughts and emotions, as these are often not directly observable. Previous studies have highlighted the limitations of relying on parent reports for emotional or psychological assessments due to these challenges (Achenbach et al. 1987; De Los Reyes and Kazdin 2005). Thus, while we made efforts to minimize recall bias by using structured and clear reporting tools, this remains a potential source of error in our study. Finally, the validation of the ASC‐ASD‐P relied on standard instruments developed for the general population, such as the CBCL and SDQ. However, as previously mentioned, a recent review (Halvorsen et al. 2024) highlights the limited validity of these measures when assessing autistic individuals. Therefore, these results should be interpreted with caution, emphasizing the need for autism‐specific instruments to accurately evaluate the overall mental health of autistic children.

In conclusion, the German version of the ASC‐ASD‐P was validated within a large clinical sample that included autistic youth often underrepresented in research, such as those with low verbal or intellectual abilities. The questionnaire exhibited good internal consistency and convergent validity, while highlighting areas for improvement to enhance construct validity. Future research should focus on validating the German self‐report version of the scale and exploring potential discrepancies between parent‐ and self‐reported assessments. Such efforts are crucial to better understand the reliability of caregiver judgments in determining anxiety symptoms.

## Author Contributions

Data were collected by Magdalena Gruner. The questionnaire was translated by Melanie Ring. The manuscript was written by Magdalena Gruner and corrected by Melanie Ring. The final manuscript was read, commented on, and approved by all authors.

## Ethics Statement

The study was approved by the ethics committee of the TUD Dresden University of Technology (ethical approval code: EK 356092018) and carried out in accordance with these regulations and the declaration of Helsinki.

## Consent

All parents of the participants gave written informed consent.

## Conflicts of Interest

The authors declare no conflicts of interest.

## Supporting information


**Data S1:** Tables.

## Data Availability

The data that support the findings of this study are available on request from the corresponding author. The data are not publicly available due to privacy or ethical restrictions.
