# Supplementary material for: Assessing Anxiety in Autistic and Non‐Autistic Youth: Validation of the German Parent Version of the Anxiety Scale for Children With Autism Spectrum Disorder
Source: Autism Res. 2025 Aug 21;18(11):2265–78. doi: 10.1002/aur.70107 (PMC12661277; doi:10.1002/aur.70107)
Supplement: Supplementary file 1 — Data S1: Tables. [file AUR-18-2265-s001.docx]

# Supplemental material

**Table S1. Comparison of ASC-ASD-P subscale ratings by group**

| Group | Subscale Comparison | *t* | *df* | *p* | *d* |
| --- | --- | --- | --- | --- | --- |
| Entire sample |  |  |  |  |  |
|  | U vs. SA | 9.63 | 316 | <.001 | 0.54 |
|  | SA vs. PA | 0.53 | 316 | .596 | 0.03 |
|  | PA vs. AA | 12.39 | 316 | <.001 | 0.70 |
| Autism group |  |  |  |  |  |
|  | U vs. SA | 8.19 | 119 | <.001 | 0.75 |
|  | SA vs. PA | 1.36 | 119 | .176 | 0.12 |
|  | PA vs. AA | 7.04 | 119 | <.001 | 0.64 |
| Non-Autism group |  |  |  |  |  |
|  | U vs. PA | 4.69 | 196 | <.001 | 0.33 |
|  | PA vs. SA | 0.34 | 196 | .731 | 0.02 |
|  | SA vs. AA | 10.12 | 196 | <.001 | 0.72 |

*Note*. U = Uncertainty Subscale, SA = Separation Anxiety Subscale, PA = Performance Anxiety Subscale, AA = Anxious Arousal Subscale, *p*-values are Bonferroni-Holm corrected.

**Table S2. Complete CBCL-scores in the autism and non-autism groups**

|  | Autism Group  (*n* = 110) | | | Non-autism Group  (*n* = 173) | | Comparison | | | |
| --- | --- | --- | --- | --- | --- | --- | --- | --- | --- |
|  | *M* | *SD* | *M* | | *SD* | *t* | df | *p* | *d* |
| CBCL subscale scores |  |  |  | |  |  |  |  |  |
| Rule Breaking | 3.2 | 2.4 | 3.0 | | 2.9 | 0.43 | 262 | 1.00 | 0.05 |
| Aggressive Behavior | 11.7 | 7.3 | 11.2 | | 8.6 | 0.50 | 260 | 1.00 | 0.06 |
| Withdrawn-Depressed | 6.7 | 3.3 | 5.4 | | 4.1 | 2.84 | 262.9 | .039 | 0.33 |
| Somatic Complaints | 2.0 | 2.5 | 1.9 | | 2.5 | 0.52 | 230.3 | 1.00 | 0.06 |
| Anxious-Depressed | 5.8 | 4.7 | 5.2 | | 4.8 | 0.95 | 234.7 | 1.00 | 0.12 |
| Social Problems | 6.1 | 3.0 | 4.2 | | 3.2 | 5.05 | 245.7 | < .001 | 0.61 |
| Thought Problems | 3.3 | 2.4 | 2.5 | | 2.6 | 2.43 | 246.2 | .111 | 0.29 |
| Attention Problems | 9.5 | 4.0 | 7.7 | | 5.1 | 3.29 | 268.5 | .010 | 0.38 |
| CBCL broad subscales |  |  |  | |  |  |  |  |  |
| Internalizing | 14.1 | 8.0 | 12.1 | | 9.2 | 1.89 | 254.4 | .353 | 0.22 |
| Externalizing | 14.9 | 9.0 | 14.2 | | 11.0 | 0.51 | 263.2 | 1.00 | 0.10 |

*Note. p*-values for subscale comparisons are Bonferroni-Holm corrected.

|  | Autism Group  (*n* = 110) | | | Non-autism Group  (*n* = 174) | | Comparison | | | |
| --- | --- | --- | --- | --- | --- | --- | --- | --- | --- |
|  | *M* | *SD* | *M* | | *SD* | *t* | df | *p* | *d* |
| SDQ total score | 17.3 | 5.1 | 14.6 | | 7.7 | 3.50 | 281.7 | < .001 | 0.39 |
| SDQ subscale scores |  |  |  | |  |  |  |  |  |
| Conduct Problems | 2.5 | 1.6 | 2.5 | | 2.0 | -0.21 | 260 | .836 | 0.02 |
| Hyperactivity-Inattention | 6.2 | 2.4 | 5.2 | | 3.2 | 3.04 | 274.9 | .008 | 0.35 |
| Emotional Symptoms | 3.07 | 2.3 | 2.8 | | 2.4 | 0.90 | 238.1 | .742 | 0.11 |
| Peer Problems | 5.6 | 2.0 | 4.1 | | 2.6 | 5.35 | 270 | < .001 | 0.62 |
| Prosocial Behavior | 4.5 | 2.3 | 5.6 | | 2.7 | -3.41 | 257.2 | .003 | 0.40 |

**Table S3. Complete SDQ-scores in the autism and non-autism groups**

*Note*. *p*-values are Bonferroni-Holm corrected

**Table S4. Correlations between ASC-ASD-P total score and subscales of AQ-Child/Adol, CBCL and SDQ**

|  | Entire sample | | Autism group | | Non-autism group | |
| --- | --- | --- | --- | --- | --- | --- |
| **AQ subscales** | *r* | *p* | *r* | *p* | *r* | *p* |
| Social Skills | .51 | <.001 | .45 | <.001 | .52 | <.001 |
| Attention Switching | .63 | <.001 | .61 | <.001 | .63 | <.001 |
| Attention to detail | .32 | <.001 | .35 | .001 | .29 | .001 |
| Communication | .43 | <.001 | .33 | .002 | .45 | <.001 |
| Imagination | .23 | .001 | .05 | 1.00 | .27 | .003 |
| **CBCL subscales** |  |  |  |  |  |  |
| Rule Breaking | .16 | .058 | .05 | 1.00 | .22 | .040 |
| Aggressive Behavior | .27 | <.001 | .13 | 1.00 | .34 | <.001 |
| Withdrawn-Depressed | .45 | <.001 | .35 | .002 | .48 | <.001 |
| Somatic Complaints | .37 | <.001 | .28 | 030 | .42 | <.001 |
| Anxious-Depressed | .71 | <.001 | .63 | <.001 | .76 | <.001 |
| Social Problems | .45 | <.001 | .28 | .028 | .52 | <.001 |
| Thought Problems | .43 | <.001 | .41 | <.001 | .42 | <.001 |
| Attention Problems | .38 | <.001 | .19 | .401 | .45 | <.001 |
| CBCL broad subscales |  |  |  |  |  |  |
| Internalizing | .66 | <.001 | .58 | <.001 | .69 | <.001 |
| Externalizing | .25 | <.001 | .12 | 1.00 | .32 | <.001 |
| **SDQ subscales** |  |  |  |  |  |  |
| Conduct Problems | .16 | .034 | -.02 | 1.00 | .26 | .003 |
| Hyperactivity-Inattention | .23 | <.001 | -.00 | 1.00 | .31 | <.001 |
| Emotional Symptoms | .71 | <.001 | .65 | <.001 | .75 | <.001 |
| Peer Problems | .30 | <.001 | .16 | .475 | .34 | <.001 |
| Prosocial Behavior | -.07 | 1.00 | .07 | 1.00 | -.11 | .748 |

*Note*. *p*-values are Bonferroni-Holm corrected

**Table S5. Correlations between ASC-ASD-subscale scores and AQ-Child/Adol subscales**

| ASC-ASD-P subscale: | AQ Social Skills | | AQ Attention Switching | | | AQ Attention to Details | | | AQ Communication | | | AQ Imagination | | |  |
| --- | --- | --- | --- | --- | --- | --- | --- | --- | --- | --- | --- | --- | --- | --- | --- |
| **Entire sample** | *r* | *p* | | *r* | *p* | | *r* | *p* | | *r* | *p* | | *r* | *p* | |
| ASC-ASD-P subscale: | | | | | | | | | | | | | | |  |
| - Separation Anxiety | .33 | <.001 | | .47 | <.001 | | .26 | <.001 | | .36 | <.001 | | .23 | <.001 | |
| - Performance Anxiety | .14 | .090 | | .25 | <.001 | | .18 | .011 | | .06 | 1.00 | | -.13 | .152 | |
| - Anxious Arousal | .33 | <.001 | | .41 | <.001 | | .20 | .005 | | .26 | <.001 | | .12 | .120 | |
| - Uncertainty | .62 | <.001 | | .69 | <.001 | | .32 | <.001 | | .51 | <.001 | | .35 | <.001 | |
| **Autism group** |  |  | |  |  | |  |  | |  |  | |  |  | |
| ASC-ASD-P subscale |  |  | |  |  | |  |  | |  |  | |  |  | |
| - Separation Anxiety | .20 | .163 | | .36 | <.001 | | .18 | .281 | | .18 | .246 | | -.04 | 1.00 | |
| - Performance Anxiety | .24 | .054 | | .44 | <.001 | | .36 | <.001 | | .17 | .320 | | -.08 | 1.00 | |
| - Anxious Arousal | .30 | .007 | | .46 | <.001 | | .26 | .026 | | .26 | .026 | | .09 | 1.00 | |
| - Uncertainty | .55 | <.001 | | .61 | <.001 | | .30 | .007 | | .38 | <.001 | | .14 | .596 | |
| **Non-Autism group** |  |  | |  |  | |  |  | |  |  | |  |  | |
| ASC-ASD-P subscale: |  |  | |  |  | |  |  | |  |  | |  |  | |
| - Separation Anxiety | .38 | <.001 | | .52 | <.001 | | .30 | <.001 | | .43 | <.001 | | .35 | <.001 | |
| - Performance Anxiety | .11 | .653 | | .19 | .074 | | .06 | 1.00 | | .03 | 1.00 | | -.16 | .179 | |
| - Anxious Arousal | .33 | <.001 | | .39 | <.001 | | .15 | .264 | | .25 | .005 | | .11 | .760 | |
| - Uncertainty | .64 | <.001 | | .71 | <.001 | | .31 | <.001 | | .54 | <.001 | | .40 | <.001 | |

*Note*. *p*-values are Bonferroni-Holm corrected.

**Table S6. Correlations between ASC-ASD-subscale scores and selected subscales of CBCL and SDQ**

|  | Entire sample | | Autism group | | Non-autism group | |
| --- | --- | --- | --- | --- | --- | --- |
|  | *r* | *p* | *r* | *p* | *r* | *p* |
| **CBCL-Anxious-depressed**  ASC-ASD-P subscale: | | | | | | |
| - Separation Anxiety | .48 | <.001 | .42 | <.001 | .51 | <.001 |
| - Performance Anx. | .65 | <.001 | .69 | <.001 | .63 | <.001 |
| - Anxious Arousal | .61 | <.001 | .50 | <.001 | .67 | <.001 |
| - Uncertainty | .56 | <.001 | .46 | <.001 | .62 | <.001 |
| **CBCL-Internalizing**  ASC-ASD-P subscale: | | | | | | |
| - Separation Anxiety | .39 | <.001 | .34 | .001 | .41 | <.001 |
| - Performance Anx. | .53 | <.001 | .56 | <.001 | .53 | <.001 |
| - Anxious Arousal | .58 | <.001 | .52 | <.001 | .61 | <.001 |
| - Uncertainty | .57 | <.001 | .46 | <.001 | .62 | <.001 |
| **SDQ-Emotional Symptoms**  ASC-ASD-P subscale: | | | | | | |
| - Separation Anxiety | .47 | <.001 | .44 | <.001 | .49 | <.001 |
| - Performance Anx. | .53 | <.001 | .55 | <.001 | .53 | <.001 |
| - Anxious Arousal | .64 | <.001 | .57 | <.001 | .67 | <.001 |
| - Uncertainty | .62 | <.001 | .54 | <.001 | .67 | <.001 |

*Note*. *p*-values are Bonferroni-Holm corrected.

**Exploratory factor analyses**

The data of the entire sample showed five eigenvalues > 1, suggesting a 5-factor-solution according to the Kaiser criterion (Kaiser, 1960). This was further supported by a parallel analysis which also indicated a number of five factors. The five factors explained 56.8% of the total variance in the items (Factor 1: 17.9%, Factor 2: 13.6%, Factor 3: 10.5%, Factor 4: 9.9%, Factor 5: 5.0%). This amount lies slightly below the threshold of 60 % which is considered as appropriate variance percentage (Hair et al., 2019). A CFA of the proposed 5-factor model was performed yielding better fit indices than the CFA of the original 4-factor structure (see Tab. S7).

**Table S7. Fit indices of the confirmatory factor analyses in the entire sample with four or five factors**

| Group | *χ^2^* | df | *χ^2^*/df | *p* | CFI^a^ | TLI^b^ | RMSEA^c^ | SRMR^d^ |
| --- | --- | --- | --- | --- | --- | --- | --- | --- |
| Original 4-factor structrue | | | | | | | | |
|  | 815.7 | 246 | 3.32 | *<*.001 | .98 | .98 | .086 [.079, .092] | .101 |
| 5-factor structure | | | | | | | | |
| Entire sample | 463.5 | 242 | 1.92 | *<*.001 | .99 | .99 | .054 [.046, .061] | .076 |

*Note*. ^a^ Comparative Fit Index, ^b^ Tucker-Lewis-Index, ^c^ Root Mean Square of Error Approximation, 90% confidence intervals in brackets, ^d^ Standardized Root Mean Square Residual

The chi-square test was again significant, but relative chi-square was below 5 and all other measures indicated an excellent model fit. A chi-square test for model comparison confirmed that the model fit significantly differed between the original 4-factor model and the proposed 5-factor model, *χ^2^*(4, *N* = 317) = 117.5, *p* *<*.001. The factor loadings are displayed in Tab. 7. Items 1 and 22 both from the Anxious Arousal Subscale had loadings below .50 on any of the factors and could be removed from the questionnaire. The exploratory factor analysis replicated the three factors Performance Anxiety (five out of five items), Anxious Arousal (six out of six items) and Uncertainty (eight out of eight items) from the original scale. However, the factor Separation Anxiety could not be replicated. Instead it was divided into two separate factors, one reflecting anxiety of being physically separated from family (now called Separation Anxiety) and a second one reflecting general worrying about the well-being of family and one-self (now called Generalized Worry). When the exploratory factor analysis was performed separately for autistic and non-autistic individuals, a 4-factor solution was preferred. However, Items 19 (“My child worries that something awful will happen to someone in the family”), 23 (“My child worries if they don’t know what will happen next e.g. if plans change”) and 24 (“My child worries that something bad will happen to him/her”) did not load on their original factor in the autism group, as well as Items 19 and 24 in the non-autism group.

**Table S8. Factor loadings of the exploratory 5-factor analysis in the entire sample**

| Items | **Uncertainty** | **Performance Anxiety** | **Anxious Arousal** | **Separation Anxiety** | **Generalized Worry** |
| --- | --- | --- | --- | --- | --- |
| 5. My child worries that people will bump into him/ her or touch him/ her in busy or crowded environments | **.65** | .19 | .19 | .06 | .14 |
| 6. My child is afraid of being in crowded places (like shopping centres, the movies, buses, busy playgrounds) in case he/ she is separated from his/ her family | **.77** | .04 | .10 | .23 | .12 |
| 9. My child is afraid of new things, or new people or new places | **.68** | .27 | .25 | .25 | -.04 |
| 10. My child is afraid of entering a room full of people | **.81** | .11 | .22 | .16 | .08 |
| 14. Feeling unsure stops my child from doing most things | **.49** | .28 | .22 | .28 | .09 |
| 16. My child always needs to be prepared before things happen | **.55** | .29 | .17 | .37 | .02 |
| 21. My child worries about being in certain places because it might be too loud, or too bright or too busy | **.77** | .12 | .15 | .24 | .09 |
| 23. My child worries if they don’t know what will happen next e.g. if plans change | **.52** | .29 | .23 | .37 | -.01 |
| 2. My child worries what other people think of him/her e.g. that he/ she is different | .00 | **.67** | .27 | -.12 | .15 |
| 4. My child feels scared when taking a test in case they make a mistake or don’t understand the questions | .20 | **.67** | .14 | .15 | .11 |
| 7. My child worries about doing badly at school work | .19 | **.72** | .12 | .05 | .04 |
| 15. My child worries when he/she thinks he/she has done poorly at something in case people judge him/ her negatively | .13 | **.76** | .19 | .09 | .15 |
| 17. My child feels afraid that he/she will make a fool of him/herself in front of people | .32 | **.71** | .23 | -.01 | .17 |
| 1. My child suddenly gets a scared feeling when there is nothing to be afraid of | .31 | .23 | **.43** | .34 | .05 |
| 3. My child’s heart suddenly starts to beat too quickly for no reason | .06 | .21 | **.67** | .15 | .07 |
| 8. My child suddenly feels so anxious he/ she feels as if he/she can't breathe when there is no reason for this | .25 | .19 | **.67** | -.06 | .04 |
| 12. When my child has a problem, he/she feels shaky | .20 | .22 | **.55** | .20 | .14 |
| 13. My child suddenly starts to tremble or shake when there is no reason for this | .15 | .16 | **.57** | .24 | .03 |
| 22. My child suddenly becomes dizzy or faint when there is no reason for this | .17 | .07 | **.42** | -.15 | .13 |
| 11. My child worries when in bed at night because he/ she does not like to be away from his/her parents/ family | .21 | -.02 | .14 | **.73** | .04 |
| 18. My child worries about being away from me | .30 | .03 | .06 | **.70** | .22 |
| 20. My child feels scared to be away from home because his/ her parents are familiar with his/ her bedtime routine | .36 | .04 | .00 | **.64** | .14 |
| 19. My child worries that something awful will happen to someone in the family | .11 | .27 | .13 | .19 | **.79** |
| 24. My child worries that something bad will happen to him/her | .17 | .30 | .29 | .18 | **.55** |

*Note*. Highest factor loadings per item are displayed in bold.

**Table S9. Factor loadings of the exploratory 5-factor analysis in the autism group**

| Items | **Uncertainty** | **Performance Anxiety** | **Anxious Arousal** | **Separation Anxiety** |
| --- | --- | --- | --- | --- |
| 5. My child worries that people will bump into him/ her or touch him/ her in busy or crowded environments | **.65** | .19 | .04 | .19 |
| 6. My child is afraid of being in crowded places (like shopping centres, the movies, buses, busy playgrounds) in case he/ she is separated from his/ her family | **.77** | .09 | .06 | .25 |
| 9. My child is afraid of new things, or new people or new places | **.66** | .19 | .46 | .11 |
| 10. My child is afraid of entering a room full of people | **.79** | .12 | .19 | .16 |
| 14. Feeling unsure stops my child from doing most things | **.45** | .36 | .22 | .22 |
| 16. My child always needs to be prepared before things happen | **.50** | .35 | .31 | .28 |
| 21. My child worries about being in certain places because it might be too loud, or too bright or too busy | **.76** | .07 | .18 | .17 |
| 2. My child worries what other people think of him/her e.g. that he/ she is different | -.08 | **.69** | .17 | .08 |
| 4. My child feels scared when taking a test in case they make a mistake or don’t understand the questions | .22 | **.56** | .34 | .08 |
| 7. My child worries about doing badly at school work | .28 | **.57** | .29 | -.07 |
| 15. My child worries when he/she thinks he/she has done poorly at something in case people judge him/ her negatively | .10 | **.77** | .22 | .04 |
| 17. My child feels afraid that he/she will make a fool of him/herself in front of people | .30 | **.77** | .21 | -.05 |
| 19. My child worries that something awful will happen to someone in the family | .14 | **.54** | -.08 | .40 |
| 24. My child worries that something bad will happen to him/her | .19 | **.56** | .03 | .39 |
| 1. My child suddenly gets a scared feeling when there is nothing to be afraid of | .28 | .21 | **.53** | .28 |
| 3. My child’s heart suddenly starts to beat too quickly for no reason | .08 | .15 | **.71** | .14 |
| 8. My child suddenly feels so anxious he/ she feels as if he/she can't breathe when there is no reason for this | .30 | .07 | **.52** | -.07 |
| 12. When my child has a problem, he/she feels shaky | .13 | .33 | **.60** | .16 |
| 13. My child suddenly starts to tremble or shake when there is no reason for this | .01 | .15 | **.46** | .26 |
| 22. My child suddenly becomes dizzy or faint when there is no reason for this | **.16** | .09 | **.16** | -.11 |
| 23. My child worries if they don’t know what will happen next e.g. if plans change | .33 | .35 | **.41** | .28 |
| 11. My child worries when in bed at night because he/ she does not like to be away from his/her parents/ family | .23 | -.03 | .26 | **.61** |
| 18. My child worries about being away from me | .16 | .16 | .15 | **.76** |
| 20. My child feels scared to be away from home because his/ her parents are familiar with his/ her bedtime routine | .33 | .10 | .13 | **.75** |

*Note*. Highest factor loadings per item are displayed in bold.

**Table S10. Factor loadings of the exploratory 5-factor analysis in the non-autism group**

| Items | **Uncertainty** | **Performance Anxiety** | **Anxious Arousal** | **Separation Anxiety** |
| --- | --- | --- | --- | --- |
| 5. My child worries that people will bump into him/ her or touch him/ her in busy or crowded environments | **.65** | .23 | .26 | .00 |
| 6. My child is afraid of being in crowded places (like shopping centres, the movies, buses, busy playgrounds) in case he/ she is separated from his/ her family | **.76** | .06 | .11 | .24 |
| 9. My child is afraid of new things, or new people or new places | **.72** | .24 | .18 | .17 |
| 10. My child is afraid of entering a room full of people | **.81** | .11 | .23 | .13 |
| 14. Feeling unsure stops my child from doing most things | **.54** | .22 | .25 | .25 |
| 16. My child always needs to be prepared before things happen | **.61** | .23 | .13 | .28 |
| 21. My child worries about being in certain places because it might be too loud, or too bright or too busy | **.76** | .16 | .16 | .27 |
| 23. My child worries if they don’t know what will happen next e.g. if plans change | **.67** | .20 | .18 | .24 |
| 2. My child worries what other people think of him/her e.g. that he/ she is different | .09 | **.68** | .35 | -.25 |
| 4. My child feels scared when taking a test in case they make a mistake or don’t understand the questions | .22 | **.73** | .08 | .14 |
| 7. My child worries about doing badly at school work | .17 | **.75** | .12 | .05 |
| 15. My child worries when he/she thinks he/she has done poorly at something in case people judge him/ her negatively | .17 | **.75** | .24 | .08 |
| 17. My child feels afraid that he/she will make a fool of him/herself in front of people | .34 | **.68** | .29 | -.01 |
| 19. My child worries that something awful will happen to someone in the family | .08 | **.37** | .29 | .35 |
| 1. My child suddenly gets a scared feeling when there is nothing to be afraid of | **.37** | .22 | **.37** | .28 |
| 3. My child’s heart suddenly starts to beat too quickly for no reason | .08 | .21 | **.65** | .13 |
| 8. My child suddenly feels so anxious he/ she feels as if he/she can't breathe when there is no reason for this | .21 | .21 | **.79** | -.07 |
| 12. When my child has a problem, he/she feels shaky | .26 | .17 | **.53** | .20 |
| 13. My child suddenly starts to tremble or shake when there is no reason for this | .29 | .13 | **.55** | .17 |
| 22. My child suddenly becomes dizzy or faint when there is no reason for this | .13 | .10 | **.59** | -.10 |
| 24. My child worries that something bad will happen to him/her | .11 | .35 | **.46** | .29 |
| 11. My child worries when in bed at night because he/ she does not like to be away from his/her parents/ family | .25 | -.03 | .12 | **.72** |
| 18. My child worries about being away from me | .41 | .02 | .06 | **.70** |
| 20. My child feels scared to be away from home because his/ her parents are familiar with his/ her bedtime routine | .41 | .03 | -.03 | **.59** |

*Note*. Highest factor loadings per item are displayed in bold.
